# Vertical Patella Fracture Fixed by Plate and Screws With Bone Graft: A Case Report

**DOI:** 10.7759/cureus.25587

**Published:** 2022-06-01

**Authors:** Khaled M Ghabban, Bashah Almustanir, Hussain M Alyassain, Siyad A Alfaraidy

**Affiliations:** 1 Orthopedic Surgery, King Salman Armed Forces Hospital, Tabuk, SAU; 2 Orthopedic Surgery, King Saud Medical City, Riyadh, SAU

**Keywords:** case report, plate and screws, articular step-off, bone graft, vertical patella fracture

## Abstract

Fractures of the patella constitute approximately 1% of all skeletal injuries. The vertical pattern represents 12-17% and the open patella fracture represents 6-30%. We here represent a rare case constituting the presence of these two uncommon (vertical type patella fracture with depression of articular surface). A 22-year-old male had a close patella fracture after a road traffic accident. The fracture was classified as a vertical and comminuted pattern. A back slab above the knee was applied and then the patient was prepared for surgery. Open reduction and internal fixation of the patella by miniplate and bone graft restore the articular surface, which reduces the risk of post-traumatic osteoarthritis. At five months postoperatively, the patient had a satisfactory joint motion with full extension and 120° of joint flexion and returned to his daily life activities without restriction. Additionally, the patient was in good health and able to bear full weight. In conclusion, the treatment choice requires a thorough knowledge of the case to ensure good stability and avoid fracture displacement.

## Introduction

The prevalence of patella fractures represents approximately 1% of all fractures, and they occur most commonly in males, with a male to female ratio of 2:1 [1]. The overall prevalence of patella fracture among male college students in Riyadh, Saudi Arabia was 23.2% and sports activities, especially soccer, were the most common cause. [2]

The pattern of patella fracture depends on the type of force and the mechanism of injury. Fractures of the patella may result from either direct or indirect mechanisms. The classic indirect mechanism is a fall on the feet in which the quadriceps eccentrically fire to decelerate the body. When the force of the fall overwhelms the resistance to knee flexion, the extensor mechanism fails.

Indirect forces typically lead to transverse fractures [3] with, at times, substantial displacement of the fracture fragments. By contrast, a direct blow more likely results in comminution, articular injury, anterior soft tissue damage, and thus open injury [4]. Combinations of patterns are common: a direct impact accompanied by knee flexion and quadriceps contraction can cause marked fragment displacement and soft tissue injury [3].

The descriptive classification of patella fractures includes nondisplaced, displaced (step-off >2-3 mm or fracture gap >1-4 mm), transverse, pole or sleeve (upper or lower), vertical, marginal, osteochondral, and comminution (stellate) [5]. The transverse fracture is the most common type, while the vertical fracture is the least common type [4].

To the best of our knowledge and based on the review of English literature (PubMed), this case is the first report of a vertical and comminuted type patella fracture treated by plate and screws with bone graft. In this report, we present a young male patient with a close right patella fracture after a road traffic accident.

## Case presentation

A 22-year-old male patient presented to the emergency room after he was in a road traffic accident complaining of right knee pain and swelling. The patient revealed no significant past medical history. His examination showed that he was conscious, alert, and vitally stable. There was moderate right knee swelling, tenderness around the right knee, especially at the lateral aspect, restricted range of motion (flexion and extension), abrasions, and the distal neurovascular examination were intact. Also, the patient was unable to bear weight on the right side.

**Figure 1:**
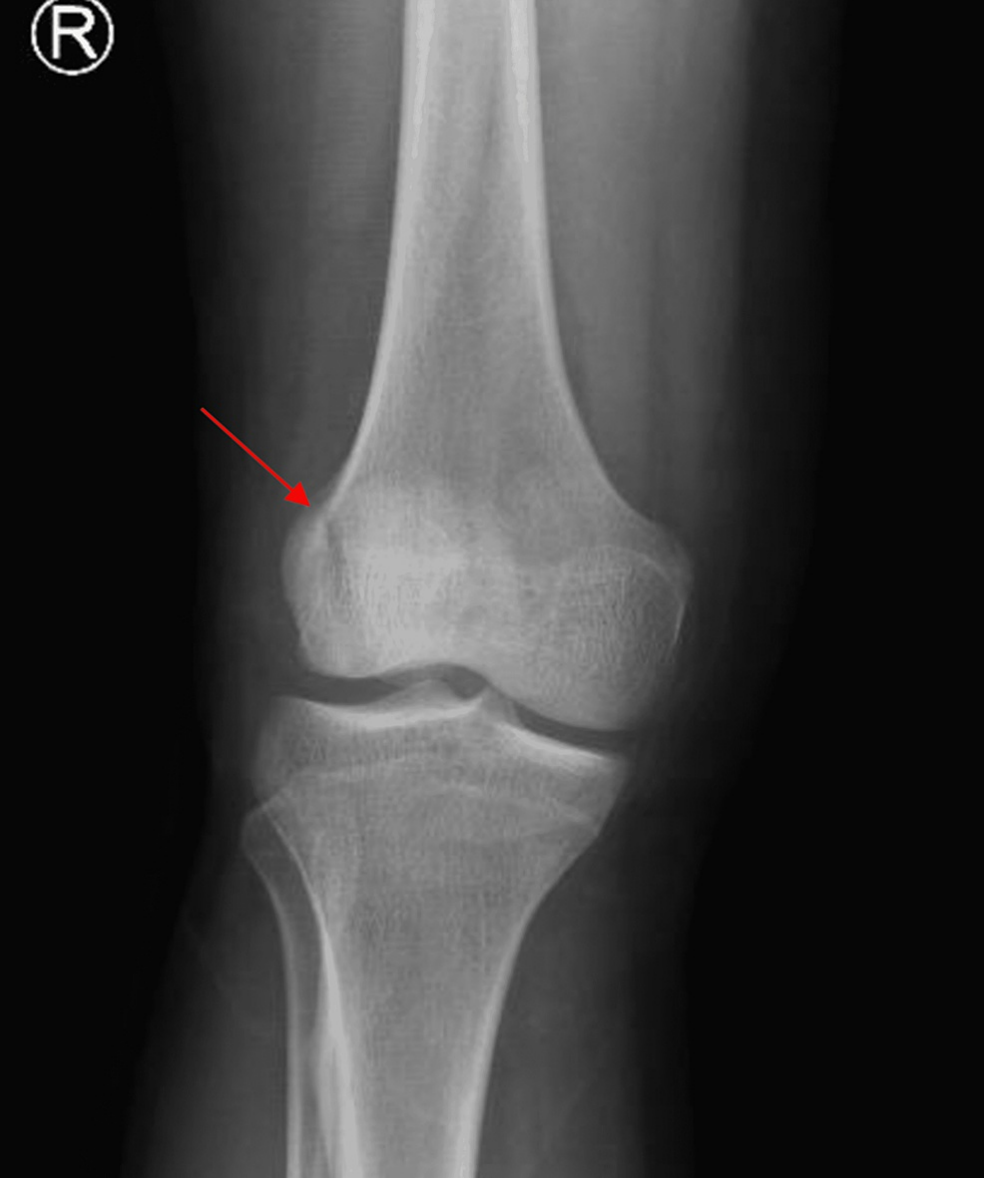
X-ray anteroposterior view of right knee showing a vertical type fracture at lateral border of patella (arrow).

**Figure 2:**
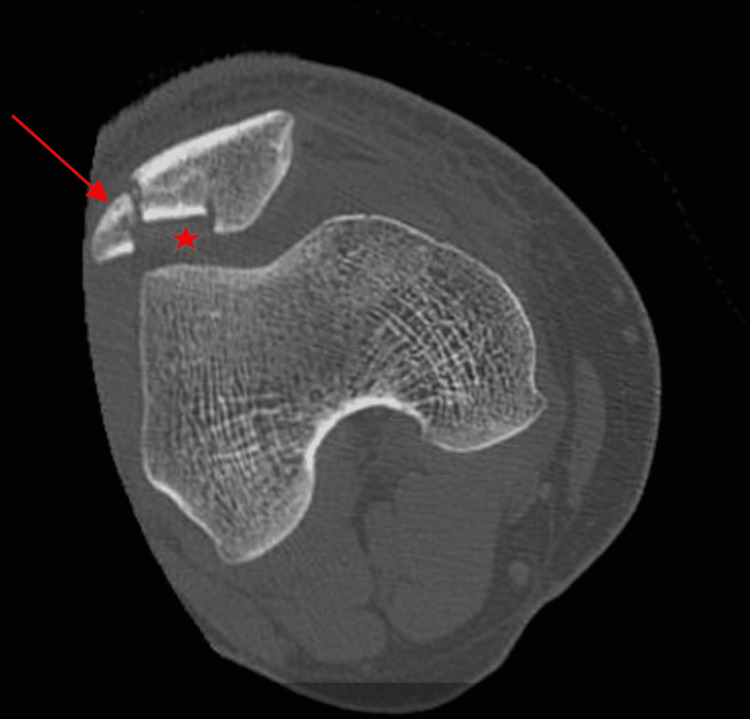
Axial CT scan of right knee showing fracture of patella at lateral border (arrow) with depression of auricular surface (asterisk).

**Figure 3:**
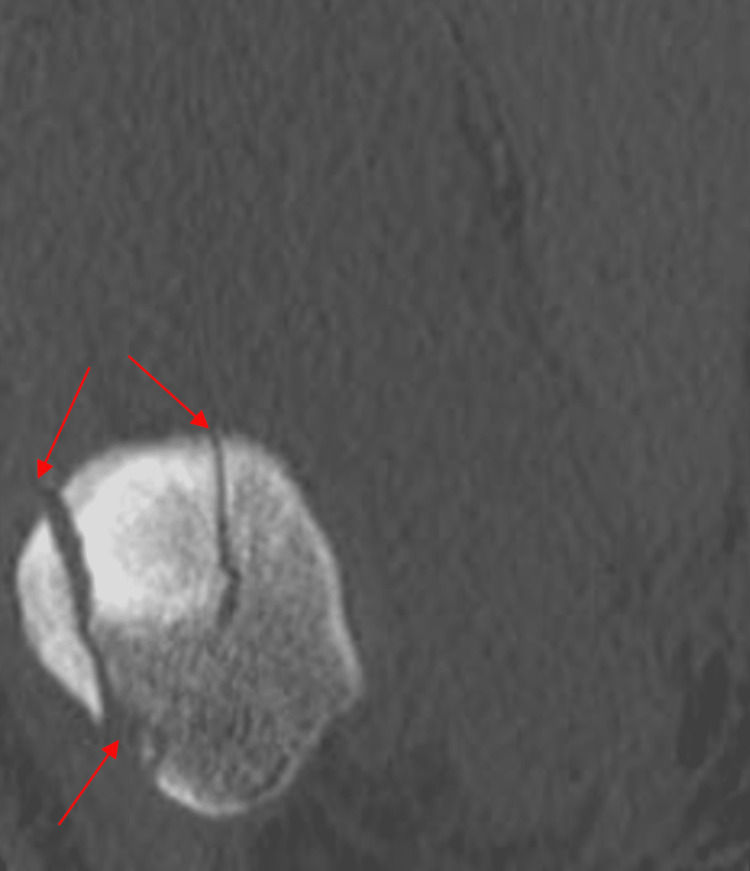
Coronal CT scan of right knee showing comminuted fracture of patella (arrows).

The anteroposterior (AP) radiograph of his right knee showed a vertical type fracture at the lateral border of the patella (Figure 1). His right knee CT scan showed a comminuted fracture of the right patella with depression of the patellar articular surface (Figures 2-3).

In the emergency room, he had an above-the-knee back slab applied with proper analgesia, and he was prepared for surgery. About seven days after the trauma, the surgery was planned to be an open reduction and internal fixation of the patella by miniplate and bone graft.

Surgical technique

**Figure 4:**
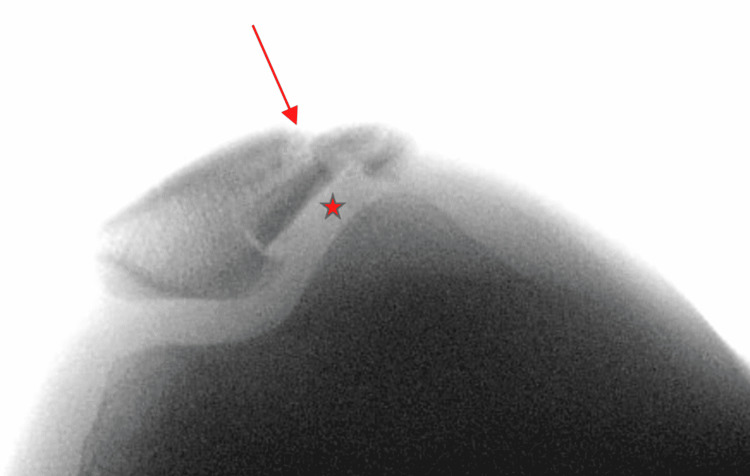
Intraoperative skyline view of right knee showing comminuted fracture mainly at lateral border of patella (arrow) with articular surface depression (asterisk).

Under general anesthesia, the patient was in a supine position with a tourniquet applied and inflated. A sandbag was inserted under the right thigh to keep the knee flexed at around 30 degrees. Before preparing and draping the AP radiograph, a lateral view and a skyline view were obtained (Figure 4).

**Figure 5:**
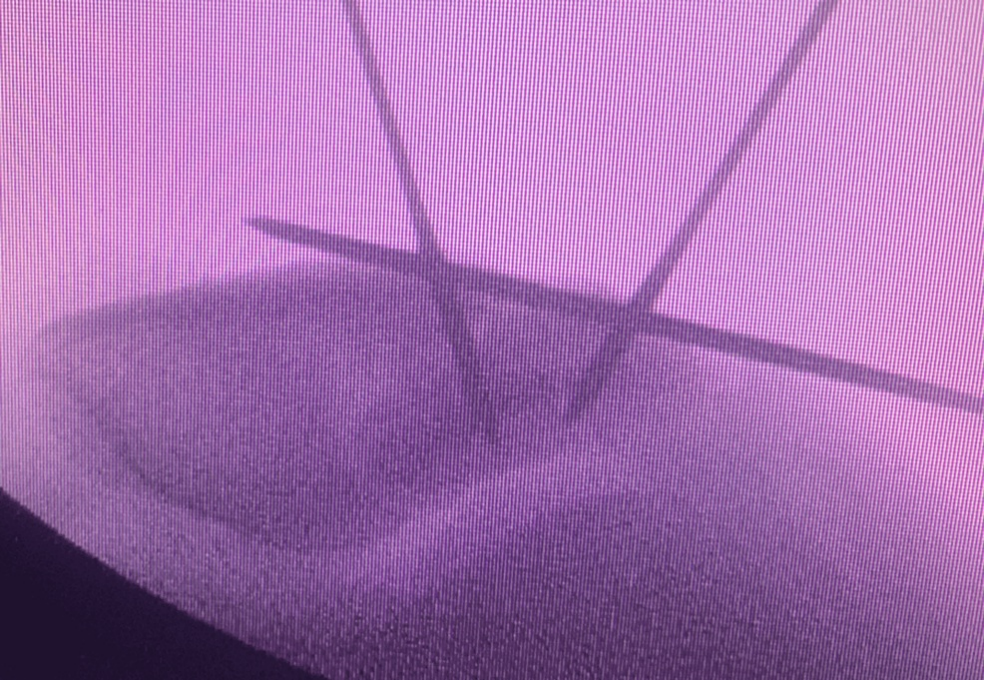
X-ray obtained after reduction and restoring of depression and filled with bone graft.

**Figure 6:**
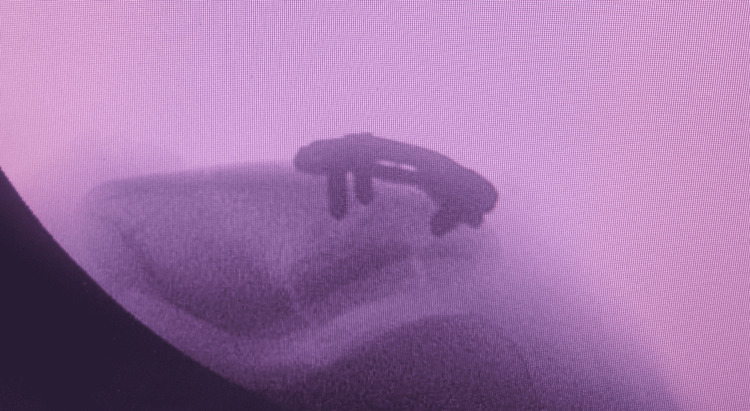
X-ray showing fixation of patella by plate and screws.

**Figure 7:**
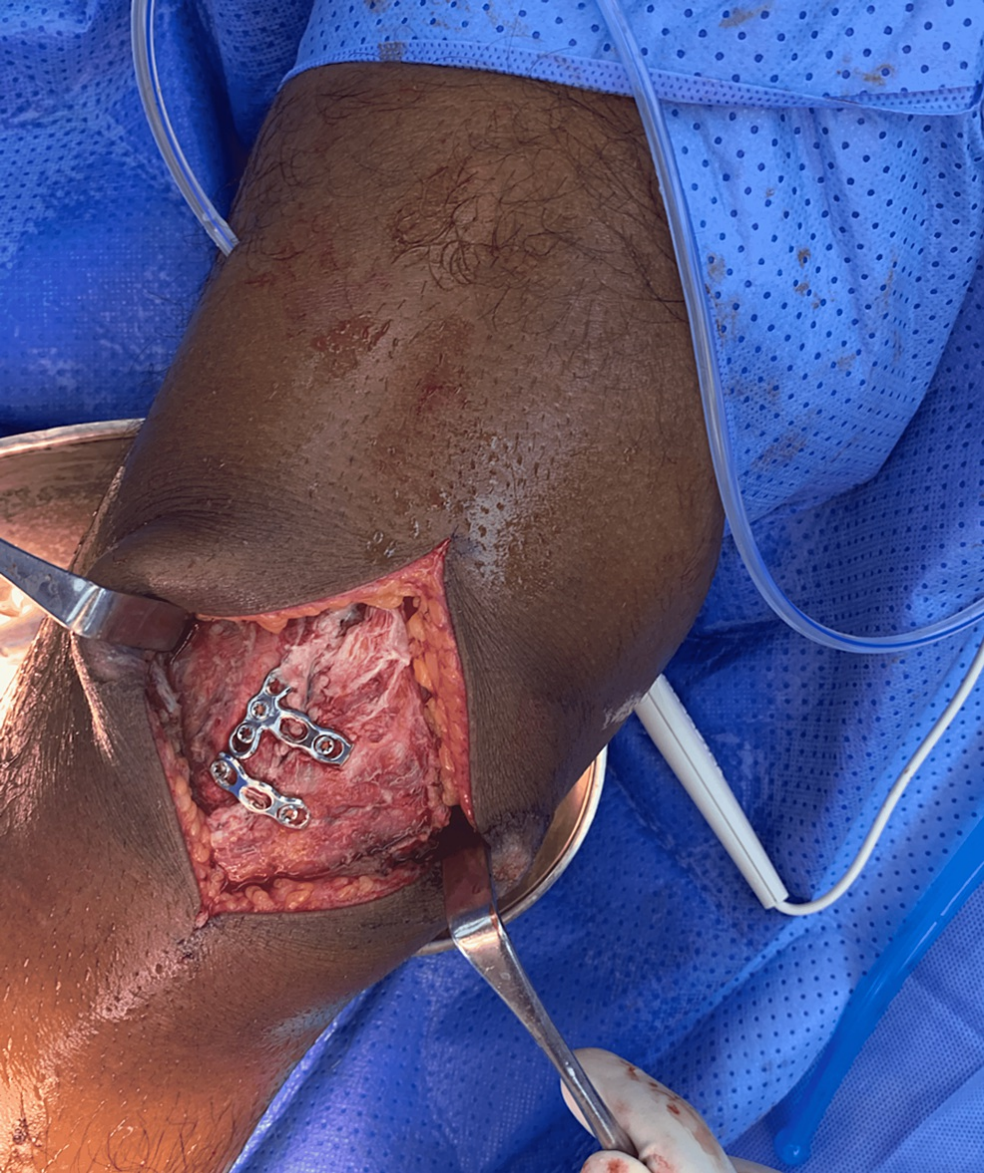
Intraoperative image post fixation.

A midline vertical incision of around 13 cm was made over the right knee. The fracture site was exposed and cleaned of debris and hematoma. The articular surface was restored by applying osteotome inside the patella to restore the articular surface, and the space was filled with bone graft. The reduction was done by small clamps and temporary K-wire fixation. The reduction was checked under the C-arm and no articular step-off was noticed (Figure 5). Fixation is done with two mini plates and screws (Figures 6-7). A dressing and a knee immobilizer were applied to him.

Outcome and follow-up

**Figure 8:**
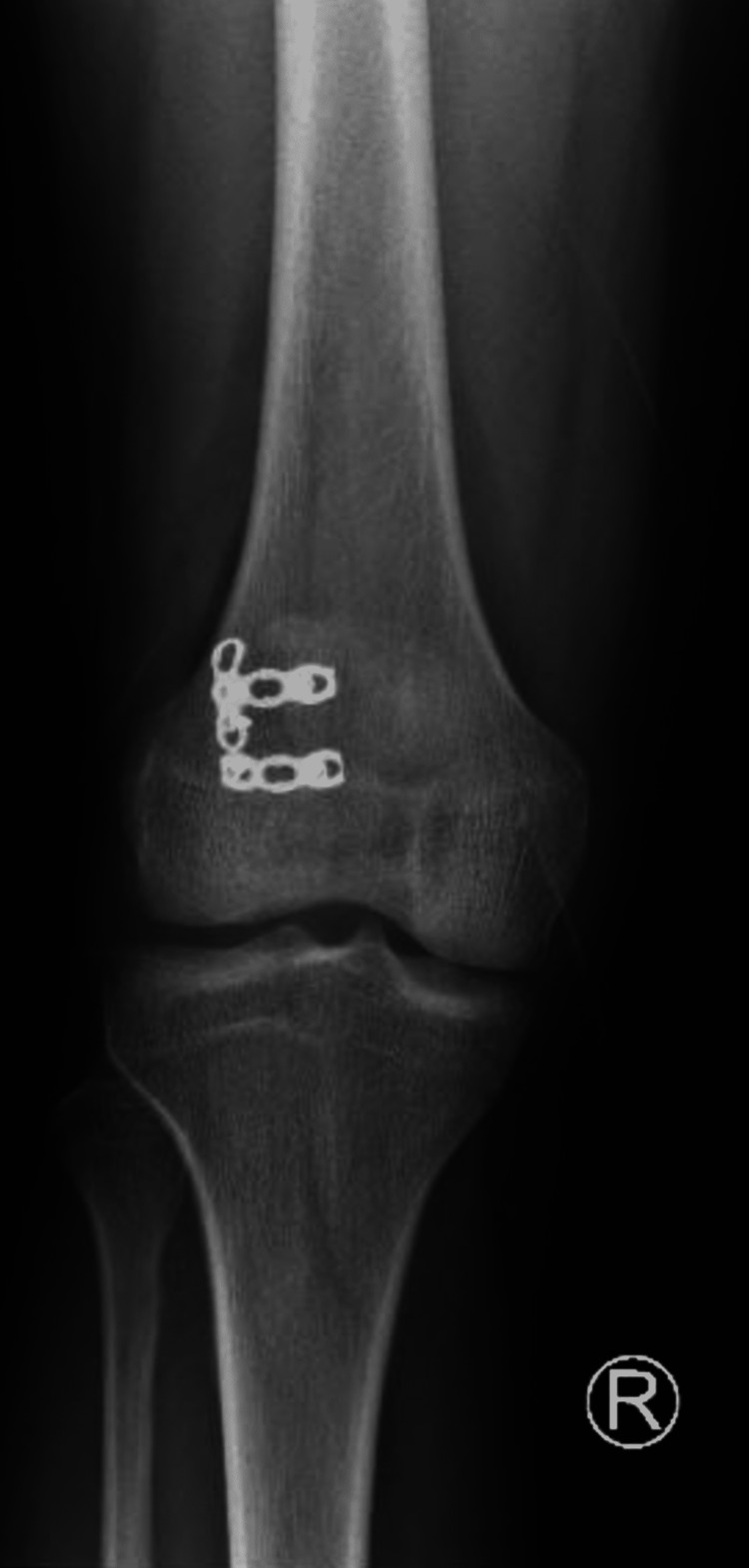
X-ray of right knee anteroposterior view at five months follow up.

**Figure 9:**
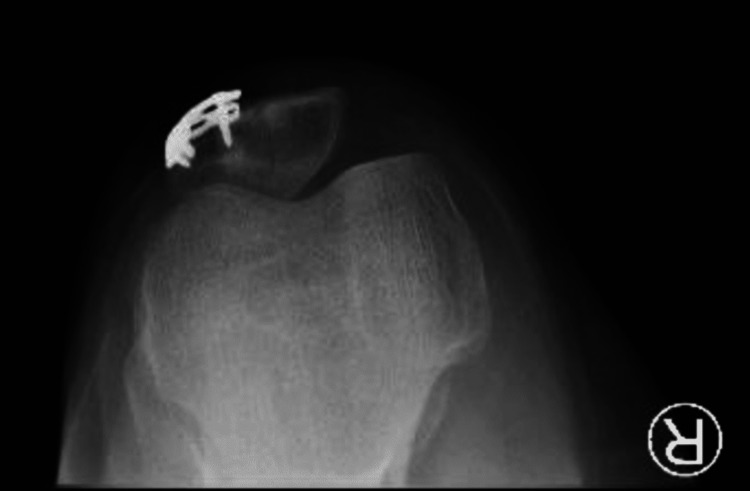
X-ray of right knee skyline view showing good healing and no signs of osteoarthritis.

He was seen in the clinic at two, four, and eight weeks. The surgical site healed well with no signs of bleeding, infection, or swelling. At five months postoperatively, the patient had a satisfactory joint motion with full extension and 120° of joint flexion and had returned to his daily life activities without restriction and instability (Figure 8-9). Additionally, the patient was in good health and able to bear full weight.

## Discussion

Vertical-type patella fractures are not common, and the incidence in male patients is higher [6]. The majority of patellar fractures result from direct trauma to the anterior side of the flexed knee [7]. Different patterns of patella fracture can also be found due to the nature of the trauma that causes the patella to fracture [8]. Likewise, this patient had comminution and vertical type fractures, which were fixed with plates, screws, and bone graft to restore the articular surface.

Patellar fractures can be managed either surgically or non-surgically [9,10]. Treatment choice is based on a detailed patient’s history, physical examination, fracture classification, and radiographs. Non-surgical management is indicated in nondisplaced fractures or displaced fractures less than 4 mm with an articular step-off of less than 2 mm and no interruption of the extensor mechanism [10]. However, surgical management is indicated for vertical, comminuted, and transverse fractures with a displacement of more than 4 mm and/or with an articular step-off of more than 2 mm, as well as an interruption of the extensor apparatus [10]. The aim of surgical treatment is to provide the support and stability of the fixation and to obtain an early range of motion.

Surgical options based on fracture mechanisms include tension band constructs, screws, or plate fixations [11]. Severe comminutions or lateral margins can be managed by patellectomy (partial or total) [3]. In intra-articular fractures, it is important to preserve the extensor mechanism and to obtain anatomical restoration of the articular surface with stable internal fixation [12]. A tension band could be the treatment of choice for transverse patella fractures [13]. Considering the fracture pattern, a vertical and comminuted patella fracture, we applied palates and screws to the fracture line and bone graft to restore the articular surface, which reduces the risk of post-traumatic osteoarthritis [12]. From a biomechanical point of view, between plates, screws, and tension bands, studies found that the application of plates and screws provides better primary stability and less fracture displacement compared to conventional tension band fixation methods [14-16]. From the above results, we conclude that fixation plates are the best treatment option for vertical-type patellar fractures. The patient’s clinical and functional final evaluation was similar to other work such as Larangeira et al. [17], although their technique used modified tension bands and wires. Functional results are determined by several factors, such as the mechanism of injury, presence of associated fractures, patient age, fracture pattern, and the proposed treatment. [6]

The patient’s functional limitation is justified by the high-energy mechanism of trauma and exposure to the fractured cartilage. We also have to consider that the fracture was vertical and comminuted at the young age of the patient, which positively influenced the final outcome.

## Conclusions

Choosing the best treatment option requires thorough clinical and radiographic examinations to rule out a ruptured extensor mechanism. It is necessary to understand the traumatic mechanism to define the type of fracture. Operative management appears to play a major role in restoring the articular surface depression. Plates and screws are the treatment of choice for patients suitable for a reconstructive procedure. Partial or total patellectomy is also an option for small fragments or where satisfactory internal fixation cannot be achieved.

The purpose of surgical treatment is to ensure anatomical reconstruction of the patella with good stability and less fracture displacement. Plates and screws with a bone graft can effectively treat vertical and comminuted patellar fractures with depression in the articular surface and offer a new strategy resulting in satisfactory results without obvious complications. It is important to emphasize that joint line position can be sufficiently restored with the use of a bone graft.
